# Preparations of Rectal Suppositories Containing Artesunate

**DOI:** 10.3390/pharmaceutics12030222

**Published:** 2020-03-02

**Authors:** Suzanne Persaud, Sandra Eid, Natalia Swiderski, Ioannis Serris, Hyunah Cho

**Affiliations:** School of Pharmacy and Health Sciences, Fairleigh Dickinson University, Florham Park, NJ 07932, USA; spersaud@student.fdu.edu (S.P.); seid@student.fdu.edu (S.E.); nswider@student.fdu.edu (N.S.); serris@student.fdu.edu (I.S.)

**Keywords:** artesunate, suppository, micelles, 3D printing

## Abstract

Rectal artesunate suppositories are a useful option for pre-referral treatment of severe malaria, specifically in children under 6 years of age in remote malaria-endemic areas. The main challenges are to improve the solubility of drugs in the rectal fluids and prevent the product from turning rancid or melting in a tropical climate. In this short proof-of-concept study, three types of rectal suppositories of artesunate were prepared: (i) polyethylene glycol (PEG)-based suppositories carrying free artesunate (non-modified artesunate), (ii) PEG-based suppositories carrying artesunate-loaded micelles and (iii) 3D-printed suppositories carrying a PEG/artesunate mixture. Physical parameters of suppositories, release profiles of artesunate (the fastest to the slowest: ii≥i>iii) and thermostability (the most stable to the least stable: iii>ii>i) of suppositories at increased temperature were assessed to determine the advantages and disadvantages of each formulation.

## 1. Introduction

Per Center for Disease Control and Prevention (CDC), malaria is a fatal disease caused by the protozoan parasite Plasmodium [1]. There are four types of Plasmodium, but the type that causes the most severe reaction is *Plasmodium falciparum*, which infects anopheles mosquitoes that feed on human blood. The occurrence of malaria is highly dependent on the climatic factors such as humidity and rainfall, with temperature being the most critical factor. This is because *P. falciparum* cannot complete its growth cycle in the anopheles mosquitoes at temperatures below 20 °C, preventing any mode of transmission. Transmission occurs most frequently where the regions are hotter and declines as the temperature decreases. The highest transmission is found south of the Sahara Desert in Africa and Papua New Guinea.

In the United States, 1700 cases of malaria are reported yearly due to travelers and immigrants returning from countries with high malaria transmission rates, such as Africa and South Asia. The treatment for severe malaria consists of intravenous (IV) antimalarial medications [2,3]. Previously, the first-line treatment was IV quinidine, but the drug was discontinued by the manufacturer in December 2017. The product distribution has ceased, and remaining products on the market were labeled expiry in March 2019. Since then, IV artesunate is now the first-line drug for treatment of severe malaria in the United States, recommended by the World Health Organization (WHO). CDC is now providing IV artesunate for the treatment of severe malaria. The WHO suggested pre-referral treatment options for the patients in areas where comprehensive treatment and care cannot be provided immediately: the recommended pre-referral treatment options are IM artesunate > rectal artesunate > IM artemether > IM quinidine.

Rectal artesunate suppositories are among the options for pre-referral treatment of severe malaria, specifically in children under 6 years of age in remote areas. Up to 80% of the children who live in rural areas die before reaching the hospital, due to a lack of resources [4]. At the community level, access to treatment (e.g., intramuscular injection (IM) artesunate) is still poor, ranging from hours to sometimes days. At this point, the disease may have progressed too far to be treated successfully. Rectal artesunate suppositories can treat young children who cannot take a medication orally due to vomiting or impaired consciousness and are pending transfer to a high-level facility where they can receive complete treatment. Per the WHO’s guideline, a single dose of 10 mg/kg body weight of artesunate should be given rectally. The most widely studied rectal artesunate suppository is Rectocaps^®^ (Mepha Pharmaceuticals, Aesch-Basel, Switzerland), formulated as 50 mg or 200 mg suppositories. Rectocaps^®^ is a rectal capsule consisting of a capsule shell (gelatin, glycerol, and titanium dioxide) and the fill blend (hard fat, medium chain triglyceride). The product shelf-life is 24 months, and Rectocaps^®^ should not be stored above 25 °C, in particular above 30 °C.

Hinton et al. studied caregivers’ acceptance of using artesunate suppositories for treating childhood malaria in Papua New Guinea [4]. After a 3 day course of artesunate suppository treatment, 99% of the caregivers agreed that the treatment was effective. Forty-eight percent of the caregivers reported that the route of administration was advantageous because it was an alternative to oral administration, which they found to be difficult in their own children. Twenty-four hours after the beginning of treatment, 94% of the child patients were afebrile and the mean reduction in parasite density from baseline was 99.3%. Gomes et al. conducted a trial in Bangladesh, Ghana, and Tanzania, in patients with suspected severe malaria who could not be treated orally [5]. They were allocated randomly to a single artesunate or placebo suppository, and then referred to clinics at which injections could be given. For the patients who did not arrive at clinics after more than 6 h post-suppository treatment, pre-referral rectal artesunate significantly reduced death or permanent disability [5]. Karunajeewa et al. compared artesunate suppositories with IM artemether, in an open-label, randomized trial in children with severe *Plasmodium falciparum* malaria in Papua New Guinea [6]. In severely ill children, artesunate suppositories were just as effective as IM artemether. The authors recommended rectal artesunate suppositories as an excellent alternative to parenteral artemisinin therapy in a rural area where injections cannot be given. Okebe et al. summarized several trials and investigated the impact of rectal artesunate as a pre-referral intervention on the mortality [7]. The authors concluded that rectal artesunate as a pre-referral intervention showed diverging effects on all-cause mortality in different age groups with severe or complicated malaria. In young children, rectal artesunate appeared to reduce the risk of death by 26%.

Artesunate is a semi-synthetic derivative of the sequiterpine lactone artesinin with antimalarial, antiviral, and potential antineoplastic properties. Upon hydrolysis of artesunate’s active endoperoxide bridge moiety by liberated heme in parasite-infected red blood cells, reactive oxygen species and carbon-centered radicals form, which have been shown to damage and kill parasitic organisms. Per the International Pharmacopoeia, artesunate is very slightly soluble in water at 25 °C. There is a small volume of the rectal fluids (ca. 1–3 mL) [8], and although artesunate is considered to be water soluble, having artesunate dissolved in the rectal fluids for absorption remains challenging. Besides artesunate’s low solubility, obtaining the thermostability of the rectal suppositories at elevated temperatures (e.g., 30 °C) is important, considering that malaria-endemic countries have extremely high temperatures.

Three-dimensional (3D) printing is a flexible prototyping technology that has the potential to revolutionize the field of drug delivery with its inherent advantages of customizability and the ability to fabricate complex solid dosage forms with high accuracy and precision [9]. Further, 3D printing can fabricate solid dosage forms with variable densities and diffusivities, complex internal geometries, multiple drugs and excipients. The 3D printing technique has become a useful tool in research and development, by reducing both time and costs in the early stage of a novel manufacturing concept. As Maulvi et al. summarized, 3D printing in the pharmaceutical industry represents a tool for designing simple, accurate, inexpensive, structured and tailored drug delivery systems [9]. This customizability allows the expansion of research and development of drug delivery systems using 3D printing. In recent years, 3D printing has been explored as precise, efficient, and customizable technology for constructing various materials in the aeronautical, health care, fashion, and biomedical industries [10]. As an additive manufacturing method, 3D printing has been used to create various biomedical and pharmaceutical materials, from medical devices corresponding to specific patient anatomy to cell-based regenerative medicine. Further, 3D printing is a unique method, with the capability to manufacture personalized dosage forms that lack in availability in the market due to a rarity of diseases or symptoms and unconventionality of drug delivery routes, for example, intraperitoneal inserts, vaginal rings, and rectal suppositories. We previously 3D printed nanogel disc rounds (12 mm in diameter × 1 mm in thickness) carrying paclitaxel and rapamycin in a convenient and consistent fashion [11]. Briefly, nanogel was 3D printed into a solid to evade premature gelation during storage and the burst release of the payloads. The 3D-printed nanogel discs permitted successful intraperitoneal delivery of paclitaxel and rapamycin in vivo in ES-2-luc ovarian cancer-bearing xenograft mice. These discs also prevented post-surgical peritoneal adhesions in the treated xenograft mice. Sun et al. 3D printed analgesic suppositories for rectal and vaginal applications [12]. The authors demonstrated that 3D printing served as a useful tool in creating personalized suppository molds to meet the demands of the physicians and patients. Tagami et al. 3D printed water-soluble suppositories for the controlled release of progesterone [13].

In this short proof-of-concept study, three types of rectal suppositories of artesunate were prepared: polyethylene glycol (PEG)-based suppositories carrying free artesunate (non-modified artesunate) were prepared using the fusion method, PEG-based suppositories carrying artesunate-loaded micelles were prepared using the lyophilization of the micelles followed by the fusion method, and core-shell suppositories consisting of a polyvinylalcohol (PVA) shell filled with a PEG/artesunate mixture were prepared using the 3D printing technique followed by the fusion method. Physical parameters of suppositories, release profiles of artesunate and thermostability of suppositories at 30 °C were assessed to determine the advantages and disadvantages of each prototype.

## 2. Materials and Methods

### 2.1. Preparation of Free-Artesunate Suppositories

Four artesunate suppositories were prepared using the fusion method. Briefly, 4 g of PEG 3350 (Fisher Scientific, Fair Lawn, NJ, USA) and 11 g of PEG 1000 (Research Products International, Mt. Prospect, IL, USA) were mixed together, melted at 60 °C on a hot plate, and cooled down to 50 °C. Then, 500 mg of artesunate (Acros Organics, Morris Plains, NJ, USA) was added to the melted PEG mixture and stirred until uniformly dispersed. The mixture was poured into four cavities of an aluminum suppository mold (Fisher scientific, Waltham, MA, USA) and allowed to solidify at room temperature (25 °C). Suppositories were removed from the mold, numbered, and measured for weights, heights, and widths. Digital caliper was used to measure heights and widths of the widest part of the suppositories.

### 2.2. Preparation of Suppositories Containing Artesunate-Loaded Micelles

Four suppositories containing artesunate-loaded micelles were prepared using the fusion method. First, polymeric micelles were prepared by vigorously vortexing 1 g (low), 1.5 g (mid) or 2 g (high) of Kolliphor P 470 (BASF, Florham Park, NJ, USA) dissolved in 10 mL of cold distilled water with 500 mg artesunate dissolved in 10 mL of *tert*-butyl alcohol (Sigma Aldrich, Allentown, PA, USA) at 60 °C. The mixture was then kept frozen for 3 days and lyophilized (0.6 mbar, −45 °C) for 1 day. The lyophilized cake of micelles carrying artesunate was added to 14, 13.5, or 13 g of the melted PEG mixture (PEG 3350:PEG 1000 = 4:11 *w*/*w*). The mixture was poured into four cavities of an aluminum suppository mold and allowed to solidify. Suppositories were removed from the mold, numbered, and measured for weights, heights, and widths.

### 2.3. Preparation of 3D-Printed Suppositories Containing Artesunate

**Figure 1:**
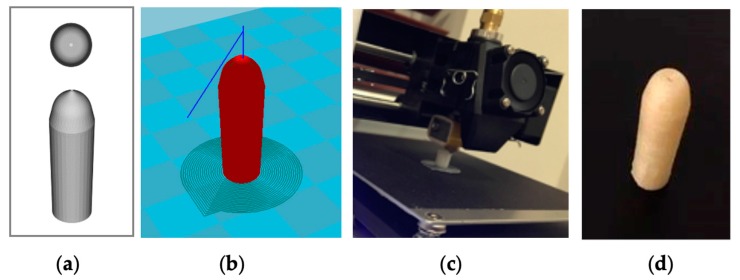
The 3D printing of a hollow suppository shell with an orifice: (**a**) Computer-aided design (CAD) of a suppository shell (top and side); (**b**) stereolithography (STL) image for 3D printing; (**c**) 3D printing of a polyvinylalcohol (PVA) shell; (**d**) a 3D-printed hollow suppository shell with an orifice on top.

Polyvinylalcohol (PVA) artesunate suppositories were 3D printed using the fused deposition modeling (FDM) technique. First, the model of a hollow suppository with an orifice on top was established with FreeCAD 0.18 (Figure 1). The CAD (computer-aided design) file was further converted into the stereolithography (STL) file (Figure 1). Using the Cura 15.04.6 software, the STL file was converted into the GCode prior to 3D printing. The 3D printing of the hollow PVA suppository shell was performed with a Monoprice MP Select Mini Pro 3D Printer (Monoprice, Inc, Rancho Cucamonga, CA), which exploits the FDM technique. The PVA filament, at a filament diameter of 1.75 mm (eSun, Shenzhen, China), was loaded for printing. A nozzle size of 0.4 mm, bed temperature of 60 °C, printing temperature of 200 °C, printing speed of 50 mm/s, traveling speed of 150 mm/s, and retraction speed of 40 mm/s were used during 3D printing. The quality parameters were set to (i) 20% infill density, (ii) 0.1 mm layer height, (iii) 0.8 mm shell thickness, and (iv) 0.6 mm bottom/top thickness. A layer of brim (single wall lines, 15% fill density, 0.7 mm distance X/Y, and 0.15 mm distance Z) was printed to support the desired object. A total of 6.4 g PEG mixture (PEG 3350:PEG 1000 = 4:11 *w*/*w*) and 500 mg artesunate (prepared as described earlier) were drawn up with a BD disposable 3 mL syringe with a luer-lok tip and a 18 gauge BD PrecisionGlide needle (BD, Franklin Lakes, NJ) and injected into as many suppositories as possible through the orifice until completely filled. Suppositories were allowed to solidify, numbered, and measured for weights, heights, and widths. The layers of the 3D-printed suppository shells were assessed using a microscope (VWR, Radnor, PA).

### 2.4. In Vitro Drug Release

Each suppository (*n* = 3 per type) of free-artesunate suppositories, suppositories containing artesunate-loaded micelles, and 3D-printed suppositories containing artesunate was immersed in 50 mL of 0.01× PBS buffer (pH 7) with a lower buffer capacity of 5.7 × 10^−5^ mol/L/pH that mimics the less effective buffer capacity of rectal fluids. The buffer was kept at 37 °C and stirred at 100 rpm. Samples (1 mL of the release medium) were taken at increments of 5 min, ranging from 0 to 40 min, and the amount of artesunate released into the buffer was measured using High-Performance Liquid Chromatography (HPLC). The release medium was replenished with fresh PBS to maintain sink conditions. The pH of the buffer was recorded during the drug release test.

The Z-average particle size of the medium samples for suppositories carrying artesunate-loaded micelles with the lower polymer content were determined by dynamic light scattering (DLS) measurements using Zetasizer Nano-ZS (Malvern Instruments, United Kingdom) at 10, 25, or 37 °C with a detection angle of 173° and a He–Ne ion laser (4 mW, λ_max_= 633 nm) for the incident beam. Autocorrelation functions were created based on the cumulant analysis using the Stokes–Einstein equation.

### 2.5. Determination of the Content of Artesunate

The amount of artesunate was determined with the 1200 Infinity Series HPLC system (Agilent, Santa Clara, CA, USA), involving the following parameters: (i) Atlantis T3 C_18_ column (3.0 × 150 mm) maintained at 25 °C, (ii) an injection volume of 10 µL, (iii) 0.1% formic acid in water (mobile phase A) and 0.1% formic acid in acetonitrile (mobile phase B), (iv) gradient elution from 40% to 60% of a mobile phase B in 10 min, (v) a flow rate of 1 mL per minute, (vi) a UV detection wavelength of 220 nm, and (vii) a retention time at 6.5 min. Based on the S.D. of response and slope, the limit of detection and the limit of quantitation were estimated to be 0.16 and 0.55 mcg/mL, respectively.

### 2.6. Effect of Termperature on Thermostability 

Free-artesunate suppositories, suppositories containing artesunate-loaded micelles, and 3D-printed suppositories containing artesunate (*n* = 3) were wrapped in foil and incubated at 30 °C (35% RH), a temperature representative of ambient conditions in tropical countries, for 6 h. A visual observation of the suppositories was made, and the weights of the suppositories and the amounts of artesunate that did not undergo degradation were assessed.

### 2.7. Statistical Analysis

For statistical analysis, ordinary one-way ANOVA with Sidak’s multiple comparison tests by GraphPad Prison ver 7.04 was used.

## 3. Results

### 3.1. Physical Properties of Suppositories Carrying Artesunate

Three types of suppositories carrying artesunate were prepared: (i) PEG-based suppositories carrying free artesunate, (ii) PEG-based suppositories carrying artesunate-loaded micelles, and (iii) 3D-printed core-shell suppositories consisting of a PVA shell filled with a PEG/artesunate mixture.

As shown in Table 1, using a fusion method, PEG-based suppositories carrying free artesunate and PEG-based suppositories carrying artesunate-loaded micelles demonstrated comparable physical appearances and artesunate contents. PEG-based suppositories carrying free artesunate were c.a. 2.46 g in weight, 31.41 mm in height, and 9.55 mm in width. The average amount of artesunate was 124 mg. PEG-based suppositories carrying artesunate-loaded micelles with 1 g (low), 1.5 g (mid), and 2 g (high) of Kolliphor P470 for micellization weighed c.a. 2.41, 2.32, and 2.26 g, respectively. Those were comparable to the PEG-based suppositories carrying free artesunate in terms of their heights (c.a. 30–31 mm) and widths (c.a. 9.32–9.48 mm). The average amount of artesunate for PEG-based suppositories carrying artesunate-loaded micelles was 123 mg.

**Figure 2:**
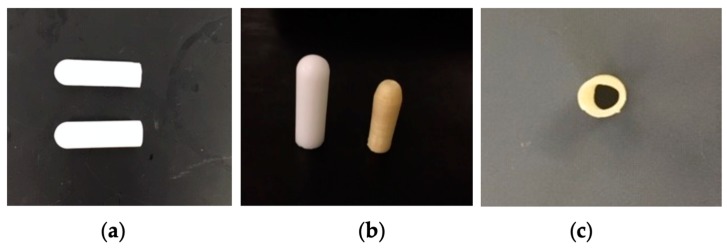
Suppositories containing artesunate: (**a**) a suppository containing artesunate-loaded micelles (low Kolliphor P470, top) and a free-artesunate suppository (bottom); (**b**) a free-artesunate suppository (left) and a 3D-printed suppository containing artesunate (right); (**c**) a cross-section of a 3D-printed suppository shell.

Water-soluble PVA suppository shells were 3D printed via the fused deposition modeling (FDM) technique (Figure 1c). Figure 1 shows a suppository shell design in CAD (Figure 1a) and an STL image for 3D printing (Figure 1b). A PVA suppository shell was c.a. 1.23 mm thick, had an orifice with an average diameter of 0.53 mm, and was hollow with a cavity width of c.a. 4.06 mm (Figure 1d, Figure 2c, Table 2). A 3D-printed PVA suppository filled with a PEG/artesunate mixture weighed 1.33 g and carried c.a. 60 mg of artesunate in its core.

### 3.2. In Vitro Drug Release

**Figure 3:**
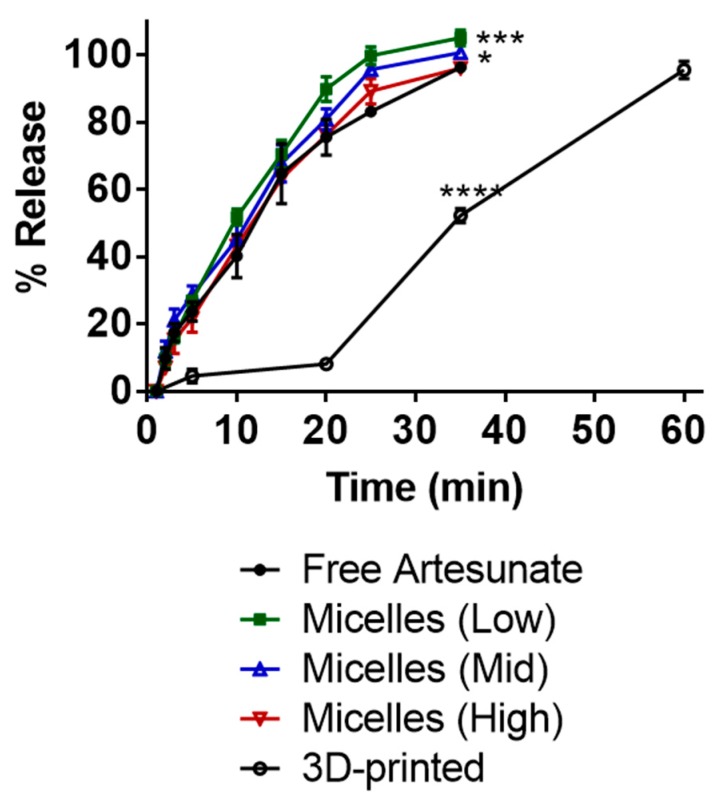
Release profiles of artesunate in PBS (pH 7, 37 °C). At 30 min, free artesunate vs. micelles (mid): * *p* = 0.0240; free artesunate vs. micelles (low): *** *p* = 0.0002; free artesunate vs. 3D printed: **** *p* < 0.0001.

Release profiles of artesunate for three types of suppositories were determined in vitro (Figure 3). The correlation coefficient (*R*^2^) obtained by using the Hixson-Crowell model had values of 0.9796, 0.9991, 0.9816, and 0.9957 for suppositories carrying free artesunate and artesunate-loaded micelles (low, mid, and high), respectively. The correlation coefficient obtained by using the Hixson-Crowell model had a value of 0.9037 for 3D-printed suppositories carrying free artesunate (Table 3). The time in minutes taken for 50% drug release (half-time) was the shortest (c.a. 9.5 min) for the PEG-based micelle (low Kolliphor P470) carrying artesunate (Table 4) and longest (c.a. 26.8 min) for 3D-printed PVA suppositories carrying artesunate.

Drug release from suppositories carrying free artesunate and those carrying artesunate-loaded micelles with high polymer content remained <96% in 35 min. Drug release from suppositories carrying artesunate-loaded micelles with low or mid polymer content reached 100% complete drug dissolution in the medium. The 3D-printed suppositories reached c.a. 50% in 35 min and c.a. 96% drug release in 60 min. Artesunate-loaded micelles released in the medium from suppositories with low polymer content had an average particle size of 63.57 ± 10.53 (PDI 0.269) at 10 min and 198.34 ± 19.85 (PDI 0.358) at 35 min. There was no statistical difference between release profiles from suppositories each carrying 124 mg free artesunate and those each carrying 63 mg of free artesunate (Figure S1).

**Figure 4:**
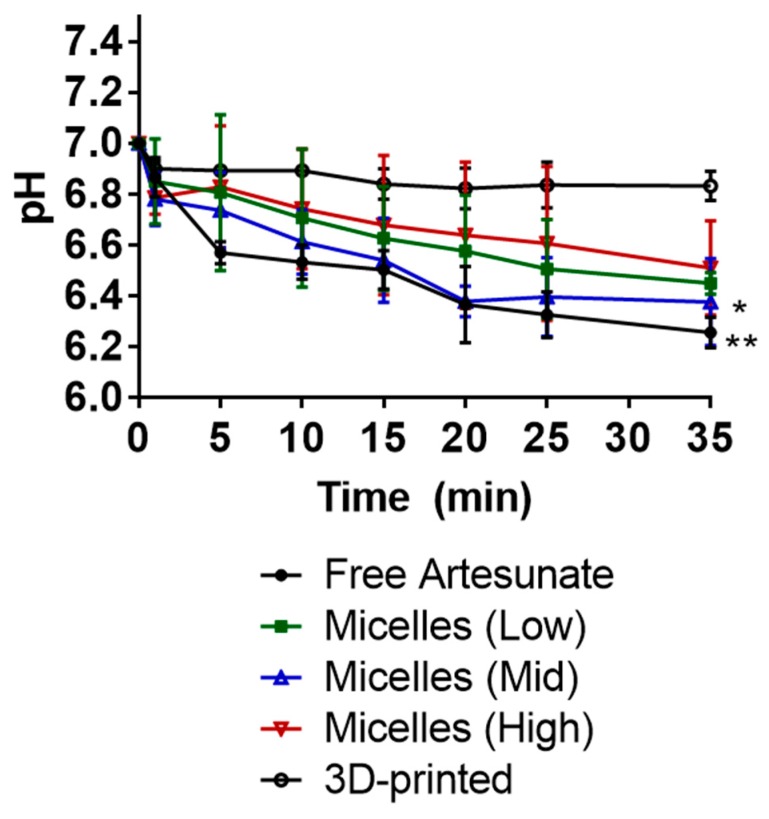
pH change in PBS (pH 7, 37 °C) medium upon drug release in vitro. At 30 min, 3D printed vs. micelles (mid): * *p* = 0.0256; 3D printed vs. free artesunate: ** *p* = 0.0063.

The pH values of the release media were determined in vitro (Figure 4). The 3D-printed suppositories carrying artesunate did not show significant effects on the acidity of the medium, decreasing the pH slightly by 0.2. Suppositories carrying artesunate-loaded micelles with low, mid, and high polymer content decreased the pH from 7 to 6.5, 6.4, and 6.5. Suppositories carrying free artesunate decreased the pH by 0.8—from 7 to 6.2.

### 3.3. Effects of Temperature on Stability of Suppositories and Artesunate

PEG-based suppositories carrying free artesunate, PEG-based suppositories carrying artesunate-loaded micelles with low polymer content, and 3D-printed suppositories carrying artesunate were incubated at 30 °C (35% RH) for 6 h. PEG-based suppositories carrying free artesunate became soft and partially dissolved at 30 °C and lost c.a. 10% drug content in 6 h (Table 5). PEG-based suppositories carrying artesunate-loaded micelles with low polymer content also became soft at 30 °C and lost c.a. 3.5% drug content in 6 h. The 3D-printed PVA shells carrying a PEG/artesunate mixture remained unchanged in their appearances and amounts of artesunate.

## 4. Discussion

On February 2018, the Indian pharmaceutical company, Cipla Ltd., received confirmation of approval from the WHO Prequalification Program for their 100 mg rectal artesunate suppositories for the pre-referral management of severe malaria [2]. This product is a rectal softgel capsule that contains hard fat (Softisan 378) and a medium chain triglyceride (Miglyol 812N). Presumably, due to the low water solubility of artesunate (56.7 mg/L at 25 °C) and the limited amount of human rectal fluids (c.a. 1–3 mL) [8], oil-based suppositories or softgel capsules containing triglycerides were used. As per the product package insert, this product should not be stored above 25 °C, in particular above 30 °C. This recommendation was made because hard fats and triglycerides in contact with moisture at room temperature easily undergo oxidation and hydrolysis reactions that cause them to turn rancid. This may prevent the products from being widely used in malaria-endemic countries with a tropical climate.

PEG is often exploited to formulate water-soluble suppositories, whereas fatty vehicles are employed to manufacture suppositories that melt at body temperature [14]. Kauss et al. used a PEG1500/PEG4000 mixture as a suppository base to prepare azithromycin suppositories for pediatric use. This suppository formulation was able to withstand a tropical climate (melting point > 50 °C). The relative bioavailability of this formulation in rabbit (c.a. 43%) was comparable to that of the oral formulation (c.a. 38%) [15].

Shinoda et al. prepared carbamazepine suppositories with Witepsol H-15, Witepsol S-55 or PEG 6000. The authors reported that PEG-based suppositories showed the most rapid drug dissolution profile in vitro (PEG > H-15 > S-55) [16]. The aqueous solubility of carbamazepine increased from 249 to 408 mg/L by adding 1% PEG in vitro. In vivo results highlighted the improved bioavailability of a rectal PEG-based suppository in rats: the bioavailability in rats was 101% for the rectal PEG-based suppository, 102% for the rectal H-15-based suppository, 79% for the rectal S-55-based suppository, and 69% for oral administration of carbamazepine.

In this study, three PEG-based suppositories were prepared: (i) PEG-based suppositories carrying free artesunate, (ii) PEG-based suppositories carrying artesunate-loaded micelles, and (iii) 3D-printed suppositories carrying a PEG/artesunate mixture (PEG 3350/PEG1000). For the second type of suppositories, the lyophilized artesunate-loaded micelles were incorporated in a PEG mixture to improve the water-solubility of artesunate. The PEG-based suppositories carrying free artesunate released slightly less than 100% (c.a. < 96%) of artesunate and dissolved in the medium within 35 min (Figure 3). Suppositories containing 1 g and 1.5 g of Kolliphor P 470 for micelle formation showed complete (c.a. 100%) artesunate dissolution in the medium. Based on the Hixson–Crowell model, the half-time was 11.2 min for PEG-based suppositories carrying free artesunate, 9.6 min for 1 g of Kolliphor P 470, and 10.2 min for 1.5 g of Kolliphor P 470. Suppositories containing 2 g of Kolliphor P 470 showed incomplete drug release in 35 min, reaching c.a. 95% release and a slightly slower half-time (11.3 min). Presumably, this was caused by the higher content of Kolliphor P 470 increasing viscosity, inducing gelation of the formulation in the medium, and as a result delaying the release of artesunate. The Hixson–Crowell model was used to calculate the half-time, as the the Hixson–Crowell model can be used to describe the sustained release of drugs from a polymeric erodible matrix that decreases in size as the system dissolves [17]. For matrix-based spherical (e.g., core-shell suppositories or suppositories carrying micelles) drug delivery systems, drug release depends on drug diffusion from the matrix and polymeric matrix erosion following polymer degradation.

As the drug release medium, 0.01× PBS buffer (pH 7) with a lower buffer capacity of 5.7 × 10^−5^ mol/L/pH was used to mimic the lower/negligible buffer capacity of human rectal fluids. Suppositories carrying free artesunate showed the most notable change in the acidity of the buffer—from 7 to 6.2 (Figure 4). Although this is not a significant change, it is presumably caused by the rapid release of free artesunate in the medium. Suppositories carrying artesunate-loaded micelles showed slight changes in pH values—from 7 to c.a. 6.5. These results imply that PEG-based suppositories carrying artesunate-loaded micelles improved the solubility of artesunate in the PBS buffer with no significant changes in the pH of the medium. Efforts have also been made elsewhere to develop nano-based drug delivery systems for systemic administration of artemisinin and its derivatives that aim to improve their poor bioavailability, short half-life in vivo, poor stability, and low solubility in water [18]—these include polymer–drug conjugates, micelles, liposomes, noisome, and carbon nanotubes. In this study, Kolliphor P 470 micelles appeared to improve the water solubility of artesunate, but the degree of improvement was not significant, as PEG itself assisted in improving the water solubility of artesunate. Although PEG-based suppositories carrying artesunate-loaded micelles performed better than fat-based suppositories at 30 °C, they still became soft on their surface and lost c.a. 3.5% of artesunate in 6 h.

To improve thermostability of artesunate suppositories in a tropical climate, we 3D-printed PVA suppository shells and inserted a PEG/artesunate mixture into the core through the orifice on top. In recent decades, patient-centric drug product development has drawn attention. Further, 3D printing has been referred to as among the most promising and fastest branches of technology being developed, meeting demands for developing patient-centric, on-demand medicine [19,20]. In our study, the FDM technique was used to extrude thermoplastic PVA filaments into a suppository shell with an orifice on top. This shell was designed to be wet prior to insertion and slowly release artesunate through the orifice upon absorption of water, followed by dissolution of PVA shells in the medium. It took 23 min to 3D print one suppository shell, and the shells were 3D printed in a consistent and convenient manner. As shown in Figure 3, less than 50% of artesunate was released in 35 min and ca. 96% of artesunate was dissolved in the medium in 60 min. The half-time was calculated to be 26.8 min by using the Hixson-Crowell model. There was no notable pH change in the drug release medium presumably due to the slow release of artesunate. There were no visual/content changes observed when 3D-printed suppositories carrying a PEG mixture/artesunate were stored at 30 °C for 6 h.

Our results indicate that a 3D-printed PVA shell is water soluble, slowly releasing artesunate over 1 h and successfully protecting artesunate from increased storage temperature. It has been reported that PVA shows a high degree of swelling in biological fluids and simulates biological tissues due to its elastic nature [21]. We envision that 3D-printed PVA suppositories carrying artesunate are among the options for pre-referral treatment of severe malaria in children under 6 years of age in remote areas with a tropical climate. The gradual release of artesunate from the shell will presumably improve artesunate’s short half-life and its low bioavailability in patients. A 3D-printed PVA shell can also be used as a promising carrier to be loaded with the oil and fatty acid suppository bases. Our future studies will focus on the feasibility of the 3D-printed PVA shells for multi-drug delivery by incorporating one drug in the shell and another drug in the core to exhibit sequential drug delivery of multi-drugs.

## 5. Conclusions

In this short proof-of-concept study, three rectal suppositories containing artesunate (PEG-based suppositories carrying free artesunate, PEG-based suppositories carrying artesunate-loaded micelles, and 3D-printed suppositories carrying artesunate) were successfully prepared. Drug release rates varied, with PEG-based suppositories carrying free artesunate being the most rapid and 3D-printed suppositories carrying artesunate being the slowest. The 3D-printed suppositories carrying artesunate showed the most optimal thermostability at 30 °C.

## Figures and Tables

**Table 1:** Physical properties of polyethylene glycol (PEG)-based suppositories carrying artesunate.

Types of Suppositories	Weight (g)	Height (mm)	Width (mm)	Artesunate (mg)
PEG based, free artesunate	2.46 ± 0.01	31.41 ± 0.05	9.55 ± 0.04	124 ± 1
PEG based, micelles (low Kolliphor P 470)	2.41 ± 0.07	31.40 ± 0.06	9.32 ± 0.11	123 ± 2
PEG based, micelles (mid Kolliphor P 470)	2.32 ± 0.06	30.19 ± 0.98	9.47 ± 0.04	123 ± 1
PEG based, micelles (high Kolliphor P 470)	2.26 ± 0.00	30.86 ± 0.39	9.48 ± 0.03	123 ± 1

**Table 2:** Physical properties of 3D-printed suppositories carrying artesunate.

Filled Weight (g)	Height (mm)	Width (Widest) (mm)	Bottom Diameter (mm)	Diameter of Orifice (mm)	Shell Thickness (mm)	Cavity Diameter (mm)	Shell Weight (g)	Artesunate (mg)
1.33 ± 0.00	24.97 ± 0.01	8.35 ± 0.09	7.45 ± 0.08	0.53 ± 0.03	1.23 ± 0.03	4.06 ± 0.07	0.71 ± 0.01	60 ± 1

**Table 3:** Comparison of the correlation coefficient (*R*^2^) for the release kinetics of artesunate using different mathematical models.

Model	PEG Based, Free Artesunate	PEG Based, Micelles (Low)	PEG Based, Micelles (Mid)	PEG Based, Micelles (High)	3D Printed
Zero order	0.9805	0.9865	0.9675	0.9913	0.9509
First order	0.9712	0.9971	0.9786	0.9917	0.8546
Higuchi	0.9790	0.9976	0.9866	0.9907	0.8382
Hixson–Crowell	0.9796	0.9991	0.9816	0.9957	0.9037
Korsmeyer–Peppas	0.9321	0.9380	0.9509	0.9344	0.6223

**Table 4:** The time (in minutes) taken for 50% drug release (half-time) using the Higuchi and Hixson-Crowell models.

Model	PEG Based, Free Artesunate	PEG Based, Micelles (Low)	PEG Based, Micelles (mid)	PEG Based, Micelles (High)	3D Printed
Higuchi	11.4	9.5	10.2	11.5	31.4
Hixson-Crowell	11.2	9.6	10.2	11.3	26.8

**Table 5:** Thermostability at 30 °C (RH 35%) for 6 h.

Types of Suppositories	% Loaded	% Remaining	Visual Appearance
PEG based, free artesunate	100 ± 0	90.0 ± 0.5	Soft/partially dissolved
PEG based, micelles (low Kolliphor P 470)	100 ± 0	96.5 ± 0.3	Soft
3D printed	100 ± 0	100 ± 0.3	No change

## Supplementary Materials

The following are available online at https://www.mdpi.com/1999-4923/12/3/222/s1, Figure S1: Release profiles of artesunate from suppositories each carrying 124 mg artesunate and those each carrying 63 mg of artesunate in PBS (pH 7, 37 °C).
