# Supplementary material for: Preparations of Rectal Suppositories Containing Artesunate
Source: Pharmaceutics. 2020 Mar 2;12(3):222. doi: 10.3390/pharmaceutics12030222 (PMC7150883; doi:10.3390/pharmaceutics12030222)
Supplement: Supplementary file 1 [file pharmaceutics-12-00222-s001.pdf]

pharmaceutics

Supplementary Materials: Preparations of Rectal Suppositories Containing Artesunate

Suzanne Persaud, Sandra Eid, Natalia Swiderski, Ioannis Serris and Hyunah Cho

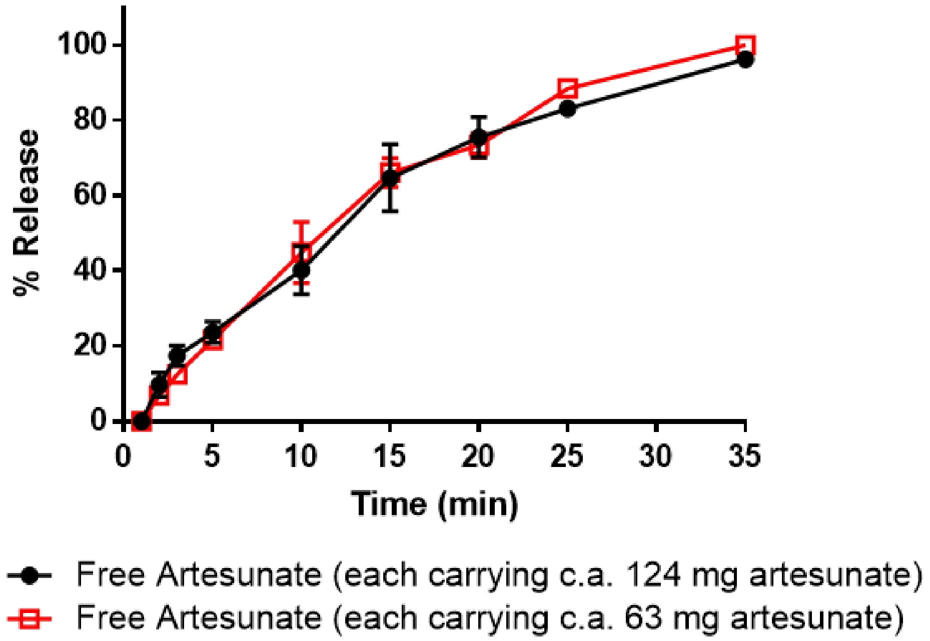

Figure S1. Release profiles of artesunate from suppositories each carrying 124 mg artesunate and those each carrying 63 mg of artesunate in PBS (pH 7, 37 °C).
